# Effects of Rocuronium on Ischemia-reperfusion Injury and Intracellular ATP Consumption: A Randomized Controlled Trial

**DOI:** 10.24546/0100504858

**Published:** 2026-06-25

**Authors:** SHINYA TAGUCHI, SHOHEI MAKINO, NORIHIKO OBATA, SATOSHI MIZOBUCHI

**Affiliations:** 1 Department of Anesthesiology, Kobe University Hospital, Kobe, Japan

**Keywords:** Rocuronium, Muscle relaxant, Ischemia, Malondialdehyde, Hypoxanthine, Adenosine triphosphate

## Abstract

**BACKGROUND:**

Muscle relaxants reduce “total” oxygen consumption, and 1.2 mg/kg of rocuronium has been shown to attenuate ischemia-reperfusion injury (IRI). Therefore, we herein investigated whether 0.9 mg/kg of rocuronium reduced “local” oxygen consumption and IRI indirectly by measuring changes in hypoxanthine (HPX), an indicator of intracellular adenosine triphosphate (ATP) consumption, and malondialdehyde (MDA), an indicator of IRI, respectively.

**METHODS:**

This study was designed as a randomized controlled trial. Female patients aged 50–79 years old who were scheduled for elective knee arthroplasty under general anesthesia were recruited. After the collection of pre-ischemia blood samples, 0.9 mg/kg of rocuronium was administered to patients in group R and a placebo to those in group C 3 minutes before tourniquet inflation. Five minutes after tourniquet deflation, post-ischemia blood samples were collected. The primary outcome was the change in the concentration of MDA (ΔMDA) after ischemia.

**RESULTS:**

Medians [interquartile ranges] for ΔMDA (μmol/L) in group R (n = 19) and group C (n = 18) were −0.47 [−1.25 to 0.13] and −0.58 [−0.67 to −0.28], respectively, with no significant difference (*p* = 0.796). Means ± standard deviations for ΔHPX (μmol/L) were 1.09 ± 0.70 and 1.71 ± 1.09 in groups R and C, respectively, and the difference between the groups was significant (*p* = 0.046).

**CONCLUSIONS:**

We were unable to confirm that 0.9 mg/kg of rocuronium attenuates IRI or postoperative quadriceps muscle weakness. While the present results suggest that rocuronium reduced intracellular ATP consumption, no clinically beneficial effects were observed.

## INTRODUCTION

Avascularization with a tourniquet is often performed in limb surgery, such as total knee arthroplasty (TKA), to reduce the amount of bleeding and ensure a bloodless surgical field. Although TKA for patients with end-stage osteoarthritis generally ameliorates pain and improves physical function [1], tourniquet use has been associated with quadriceps muscle weakness lasting 3 months [2]. In addition to orthopedic surgery, there are a number of conditions under which muscle tissue becomes ischemic, such as cardiovascular surgery and free flap surgery, and long-term ischemia results in muscle fiber necrosis and degeneration [3]. To prevent ischemic complications, the duration of ischemia needs to be minimized; however, there are currently no clear criteria and it may still exceed the planned time depending on the progress of the surgery. Even in such a case, the suppression of oxygen consumption by ischemic tissue may extend the life span of muscle cells, thereby reducing postoperative complications and preventing delays in functional recovery.

Muscle relaxants are sometimes used to reduce oxygen consumption, and have been shown to reduce “total” oxygen consumption in mechanically ventilated patients [4, 5]. However, since these effects may be due to improvements in ventilator synchrony and reduced respiratory effort, there is no evidence to show that muscle relaxants reduce “local” oxygen consumption. In 2018, Ledowski et al. [6] reported that rocuronium, a non-depolarizing muscle relaxant, exerted protective effects against ischemia in skeletal muscle in rats, and speculated that these effects were due to muscle relaxants reducing cellular oxygen consumption. Chen et al. [7] subsequently conducted a clinical study and indicated that rocuronium exerted dose-dependent protective effects against ischemia in skeletal muscle. They examined rocuronium at two doses, 1.2 and 0.6 mg/kg, and showed that only 1.2 mg/kg attenuated ischemia-reperfusion injury (IRI). However, their findings suggested that rocuronium exerted protective effects on skeletal muscle, even at 0.6 mg/kg; therefore, we suspected that other factors may have contributed to these effects. Furthermore, the maximum dose of rocuronium approved in Japan is 0.9 mg/kg, and it is unclear whether this dose effectively attenuates IRI. Accordingly, we hypothesized that 0.9 mg/kg of rocuronium attenuates IRI, and conducted the present study with the aim of confirming that this effect is due to a reduction in oxygen consumption by muscle cells. We herein investigated whether 0.9 mg/kg of rocuronium attenuated IRI and reduced “local” oxygen consumption indirectly by measuring changes in malondialdehyde (MDA), an indicator of IRI [8], and hypoxanthine (HPX), an indicator of hypoxia [9, 10], respectively. We also attempted to elucidate the mechanisms by which rocuronium protects muscle cells against ischemia and contributes to improved surgical outcomes and postoperative functional recovery.

## MATERIALS AND METHODS

### Ethics and registration

This study was designed as a randomized, single-blind, placebo-controlled, parallel-group trial performed at Kobe University Hospital. We report this trial in accordance with the Consolidated Standards of Reporting Trials (CONSORT) guidelines. The trial was approved by the Ethics Committee at Kobe University Hospital (Kobe, Japan) on July 19, 2019. The protocol was registered before starting patient enrolment in the University hospital Medical Information Network Clinical Trial Registry (UMIN000036931 on July 26, 2019). All eligible and consenting patients provided their signed, written informed consent before randomization.

### Study population

Inclusion criteria included female patients aged 50–79 years old who were scheduled for elective unilateral TKA or unicompartmental knee arthroplasty under general anesthesia from July 2019 to March 2023.

Exclusion criteria included patients with preoperative alanine aminotransferase >100 U/L; creatinine >1.5 mg/dL; a history of neuromuscular disease, drug sensitivity, or allergy to muscle relaxants; patients who were pregnant or breast-feeding; and patients taking a calcium blocker, β blocker, steroids, or other medications known to affect the action of muscle relaxants (monoamine oxidase inhibitor, phenytoin, carbamazepine, cimetidine, quinidine, metronidazole, aminoglycoside antibiotics, lincomycin antibiotics, polypeptide antibiotics, lithium, calcium, magnesium, potassium, furosemide, and thiazide diuretics) on the day of surgery.

### Randomization

The investigator enrolled patients after obtaining their written informed consent. Patients were then randomly assigned to a rocuronium group (group R) or control group (group C) by an envelope method at a ratio of 1:1. Envelopes were generated, selected, and opened by non-medical personnel not involved in the study. Patients and surgeons were blinded to the allocation results.

### Intervention

None of the patients were premedicated. An intravenous catheter was inserted into an upper extremity vein, and standard monitoring (electrocardiogram, non-invasive blood pressure, and pulse oximetry) was performed. After the administration of a preoperative prophylactic antibiotic (1 g of cefazolin or cefotiam), normal saline infusion was started. After preoxygenation, general anesthesia was induced with propofol (2–3 mg/kg), fentanyl (1–2 μg/kg), and remifentanil (0.05–0.2 μg/kg/min). Airway management was conducted using a supraglottic device. Each patient was mechanically ventilated with an inspired oxygen fraction of 0.4, and target end-tidal carbon dioxide partial pressure was 35–45 mmHg because respiratory pH changes may affect the action of rocuronium [11, 12]. Nerve block and local anesthesia were not performed prior to surgery. Anesthesia was maintained with sevoflurane (1–1.5%) and remifentanil (0.05–0.5 μg/kg/min). Propofol was not used for the maintenance of anesthesia because it has been reported to suppress increases in MDA [13]. To stabilize blood pressure and heart rate, ephedrine, phenylephrine, atropine, nicardipine, and fentanyl were used as required. After a pre-ischemia venous blood sample had been obtained, 0.9 mg/kg of rocuronium bromide was administered to patients in group R and a placebo (same volume of normal saline) to those in group C 3 minutes before tourniquet inflation. Simultaneously with tourniquet inflation, muscle contractions of the adductor pollicis induced by a train-of-four (TOF) stimulation of the ulnar nerve at 50 mA were measured by acceleromyography (TOF Watch^TM^ SX; MSD, Tokyo, Japan). After tourniquet inflation (250 mmHg), the use of rocuronium was permitted in both groups as needed. Five minutes after tourniquet deflation, a post-ischemia venous blood sample was collected. MDA, HPX, lactate ion, myoglobin, creatine kinase (CK), aspartate aminotransferase (AST), potassium, and uric acid (UA) concentrations were measured from blood samples taken before and after ischemia, and changes in these concentrations were compared in the two groups. Although lactate dehydrogenase was measured at the start of the study, the measurement method was changed during the study and, thus, it was excluded. At the end of the surgery, the surgeon administered analgesics (0.75% ropivacaine 20 ml, morphine 10 mg, dexamethasone 3.3 mg, adrenaline 0.3 mg, and normal saline 40 ml) for postoperative pain relief and 10% tranexamic acid 20 ml to reduce postoperative bleeding via a periarticular injection. Before extubation, patients who had received rocuronium were given sugammadex. Study methods for each group are shown in Fig. 1.

### Outcomes

The primary outcome of this study was the change in the concentration of MDA (ΔMDA) after ischemia. Secondary outcomes were changes in HPX, lactate ion, myoglobin, CK, AST, potassium, and UA concentrations after ischemia and changes in quadriceps muscle strength after surgery. We also investigated the relationship between the total dose of rocuronium and changes in various blood test results, as well as the number of days until discharge.

#### Measurement of plasma MDA

Blood samples for MDA measurements were divided into an EDTA-2K collection tube, centrifuged (2,000 × *g*, 5 min), and 0.05% butylated hydroxytoluene was added as an antioxidant to the resulting supernatant, which was then stored at −80°C. To measure plasma MDA concentrations, the thiobarbituric acid reactive substance (TBARS) assay was performed using the OxiSelect^TM^ TBARS Assay Kit (Cell Biolabs, Inc., San Diego, USA). One hundred microliters of sodium dodecyl sulfate lysis solution was added to 100 μL of each MDA standard and samples, mixed thoroughly, and incubated at room temperature for 5 minutes. Two hundred and fifty microliters of thiobarbituric acid reagent was added to the solution, which was incubated at 95°C for 50 minutes, cooled to room temperature in an ice bath for 5 minutes, and centrifuged at 3,000 rpm for 15 minutes. To prevent the interference of hemoglobin and its derivatives, 300 μL of n-butanol was added to 300 μL of the supernatant, which was vortexed vigorously for 1.5 minutes and then centrifuged at 10,000 × *g* for 5 minutes. After an incubation at room temperature for 10 minutes, the supernatant was used for spectrophotometric measurements. The absorbance of MDA standards and samples was read at 532 nm.

#### Measurement of plasma HPX

A previous study demonstrated that plasma HPX concentrations increased in a time-dependent manner after blood collection [14]. Blood samples for HPX measurements were divided into a PAX gene^TM^ DNA collection tube (Becton, Dickinson and Company, USA), which has been shown to prevent increases in HPX [15], centrifuged (2,000 × *g*, 5 min), and the resulting supernatant was stored at −80°C. Samples were submitted to Mie Research Laboratories (Sanwa Kagaku Kenkyusho, Mie, Japan) and analyzed.

#### Measurement of blood lactate ions

Blood samples for lactate ion measurements were divided into a heparinized tube and lactate ion concentrations were assessed using ABL800 FLEX^TM^ (Radiometer, Copenhagen, Denmark).

#### Measurement of other parameters

Blood samples for myoglobin, CK, AST, potassium, and UA measurements were divided into plain collection tubes, centrifuged (2,000 × *g*, 5 min), and the resulting supernatant was stored at 4°C. Samples were submitted to a clinical testing company (BML, INC. Japan) and analyzed.

#### Quadriceps muscle strength

Quadriceps muscle strength was measured using a hand-held dynamometer (ergoFET^TM^; Hoggan Scientific, Salt Lake City, USA) by physical therapists who were blinded to the allocation group on the day before surgery and before discharge, and the degree of change, the number of postoperative days (POD) until the measurement, and the number of POD until discharge were compared in the two groups.

### Sample size and statistical analysis

Based on the findings reported for the inhalation anesthesia group in Turan’s study [13] on tourniquet-induced IRI in the lower extremities, we estimated the mean increase in MDA to be 1.4 μmol/L and the standard deviation (SD) to be 0.4 μmol/L. Assuming that a t-test was conducted with the suppressive effects of rocuronium at 30% (0.42 μmol/L), a significance level of 5%, and a power of 90%, it was calculated that 20 patients were required in each group. In anticipation of a 15% loss from potential technical failures, we increased the target total sample size to 46.

Data for patient demographics are presented as medians [interquartile range (IQR)] or numbers. We used the Mann-Whitney *U* test for continuous data and the chi-square test for categorical data. Regarding research results, parametric data are presented as means ± SD and non-parametric data as medians [IQR]. We used the Student’s *t*-test for parametric data and the Mann-Whitney *U* test for non-parametric data. The Shapiro-Wilk test was performed to investigate whether continuous data conformed to a normal distribution. Spearman’s correlation analysis was employed to examine the relationship between the total dose of rocuronium and each item. A *p* value < 0.05 was considered to be significant. All statistical analyses were performed using EZR version 1.61 (Saitama Medical Center, Jichi Medical University, Saitama, Japan), R-based analysis software [16].

## RESULTS

### Participants

Forty-three patients were enrolled in the present study between July 2019 and May 2022, and 37 (19 in group R and 18 in group C) were included in the primary outcome analysis (Fig. 2); six were excluded from the study because of tourniquet failure (n = 2), delayed tourniquet inflation (n = 1), delayed blood collection (n = 1), blood hemolysis (n = 1), and tracheal intubation (n = 1). This study was terminated before reaching the target sample size due to the expiration of the period approved by the Ethics Committee. Baseline characteristics in the two groups are shown in Table I and Appendix 1. Patient demographics were similar between the groups. A comparison of the amounts of drugs used for anesthesia is shown in Appendix 2. Significant differences were observed in the amounts of rocuronium and sugammadex administered.

The TOF count measured simultaneously with tourniquet inflation was 0 in all patients, except for one (2 counts) in group R, confirming that ischemia had begun under the effects of rocuronium. The TOF count was 4 in all patients in group C.

### Primary outcome

Medians [IQR] for ΔMDA (μmol/L) in group R (n = 19) and group C (n = 18) were −0.47 [−1.25 to 0.13] and −0.58 [−0.67 to −0.28], respectively, with no significant difference (*p* = 0.796) (Table II and Appendix 3). Furthermore, no significant differences were observed in pre- or post-ischemic MDA concentrations between groups R and C (Fig. 3 and Appendix 4).

### Secondary outcomes

Means ± SD for ΔHPX (μmol/L) in group R (n = 19) and group C (n = 18) were 1.09 ± 0.70 and 1.71 ± 1.09, respectively, with a significant difference (*p* = 0.046) (Table II and Appendix 3). Although pre- and post-ischemic HPX concentrations did not significantly differ between groups R and C (Fig. 4a), the degree of change was significantly smaller in group R (Fig. 4b). HPX is an intermediate product of adenosine triphosphate (ATP) metabolism and is metabolized by xanthine oxidoreductase (XOR) [17] (Fig. 5), and none of the patients in this study had received an ATP or XOR inhibitor (allopurinol, febuxostat, and topiroxostat) on the day of surgery. No significant differences were noted in changes in lactate ion, myoglobin, CK, AST, potassium, or UA concentrations after ischemia (Table II and Appendix 3).

The total dose of rocuronium was weakly associated with changes in myoglobin and CK concentrations (Appendix 5, Appendix 6). However, CK concentrations were lower after ischemia in most patients, indicating that they did not reflect the effects of ischemia; therefore, this result is considered to have no clinical significance.

Means ± SD for Δquadriceps muscle strength (N) in group R (n = 16) and group C (n = 15) were −82.3 ± 43.2 and −54.3 ± 47.1, respectively, with no significant difference (*p* = 0.094) (Table III and Appendix 7).

## DISCUSSION

MDA, an intermediate product of lipid peroxidation, is an indicator of IRI [8]. In the present study, no increase was observed in the concentration of MDA post-ischemia. None of the patients in the present study took α-tocopherol (vitamin E) on the day of surgery, which may inhibit lipid peroxidation [18]. In addition, propofol, which inhibits lipid peroxidation [13], was not used to maintain anesthesia. After the completion of the present study, we found a study showing that remifentanil inhibited lipid peroxidation [19]; therefore, the effects of IRI may not have been evaluated appropriately. Previous studies reported increases in the concentration of MDA before reperfusion [13, 20] and 60 seconds [21], 15 minutes [22], and 30 minutes [7] after reperfusion. In the present study, the concentration of MDA was measured 5 minutes after reperfusion; however, it is possible that the increase in MDA was inhibited by the effects of remifentanil. If measurements had been taken 30 minutes after reperfusion, the same timing as that reported by Chen et al. [7] using remifentanil, an increase may have been observed. Therefore, further studies without the use of remifentanil are needed to confirm whether 0.9 mg/kg of rocuronium is effective against IRI. Moreover, the concentration of MDA was measured in peripheral venous blood in the present study. Since MDA concentrations were previously shown to be significantly higher in venous blood downstream of ischemic organs than in arterial blood [22], it may have been more appropriate to measure MDA in femoral vein blood. However, MDA concentrations have been assessed using arterial blood [7] and peripheral venous blood [13, 20], and both are diluted by systemic blood; therefore, its concentration is not considered to significantly differ between these samples. Furthermore, if the change in the concentration of MDA is small, it may not be detectable in diluted blood.

On the other hand, a significant inhibitory effect on the increase in HPX was observed in group R. HPX, an intermediate product of ATP metabolism, is an indicator of hypoxia [9, 10]. When the oxygen supply is sufficient relative to ATP consumption, ATP is regenerated from adenosine diphosphate (ADP) by mitochondrial oxidative phosphorylation, whereas when the oxygen supply is inadequate, ATP metabolism proceeds and plasma HPX increases [9, 23, 24] (Fig. 5). Therefore, intracellular ATP depletion may be indirectly measured from an increase in the concentration of extracellular HPX [9]. In the present study, the increase in HPX was inhibited in group R, which suggests that the pre-administration of rocuronium reduced intracellular ATP consumption, possibly by suppressing muscle contractions.

Dystrophin is a sarcolemmal membrane protein in skeletal and cardiac muscles that protects muscle cells from the mechanical stress of muscle contractions [25–28]. Ischemia results in the redistribution of dystrophin from the membrane to myofibrils [27], which is related to a decrease in mitochondrial ATP generation [28]. Previous studies demonstrated that the administration of rocuronium before ischemia inhibited the loss of dystrophin from the membrane [6, 7]. Collectively, these findings and the present results indicate that the administration of rocuronium before ischemia inhibits the ischemic redistribution of dystrophin by reducing intracellular ATP consumption.

There are several limitations that need to be addressed. The number of patients in the present study did not reach the target number. Therefore, the results obtained for the primary outcome, ΔMDA after ischemia, may not be accurate. Furthermore, remifentanil, which inhibits lipid peroxidation [19], was used in this study and, thus, the effects of IRI may not have been evaluated appropriately. In addition, the concentrations of each test item in the present study were measured 5 minutes after reperfusion; however, since the timing of increases may have differed for each item, the use of 5 minutes after reperfusion for all tests may not have been appropriate. Nevertheless, the decrease in postoperative quadriceps muscle strength was greater in group R, suggesting that the protective effects of rocuronium on skeletal muscle cannot be overly relied upon. Even if rocuronium is effective for preventing IRI and reducing oxygen consumption, the duration of these effects may be limited. Since the duration of ischemia in the present study may not have been adequate to accurately evaluate these effects, further studies, such as those in which time periods with a limited duration of ischemia are examined, are warranted.

In conclusion, we were unable to confirm that 0.9 mg/kg of rocuronium attenuates IRI or postoperative quadriceps muscle weakness. While the present results suggest that rocuronium reduced intracellular ATP consumption, no clinically beneficial effects were observed.

## Figures and Tables

**Fig. 1:**
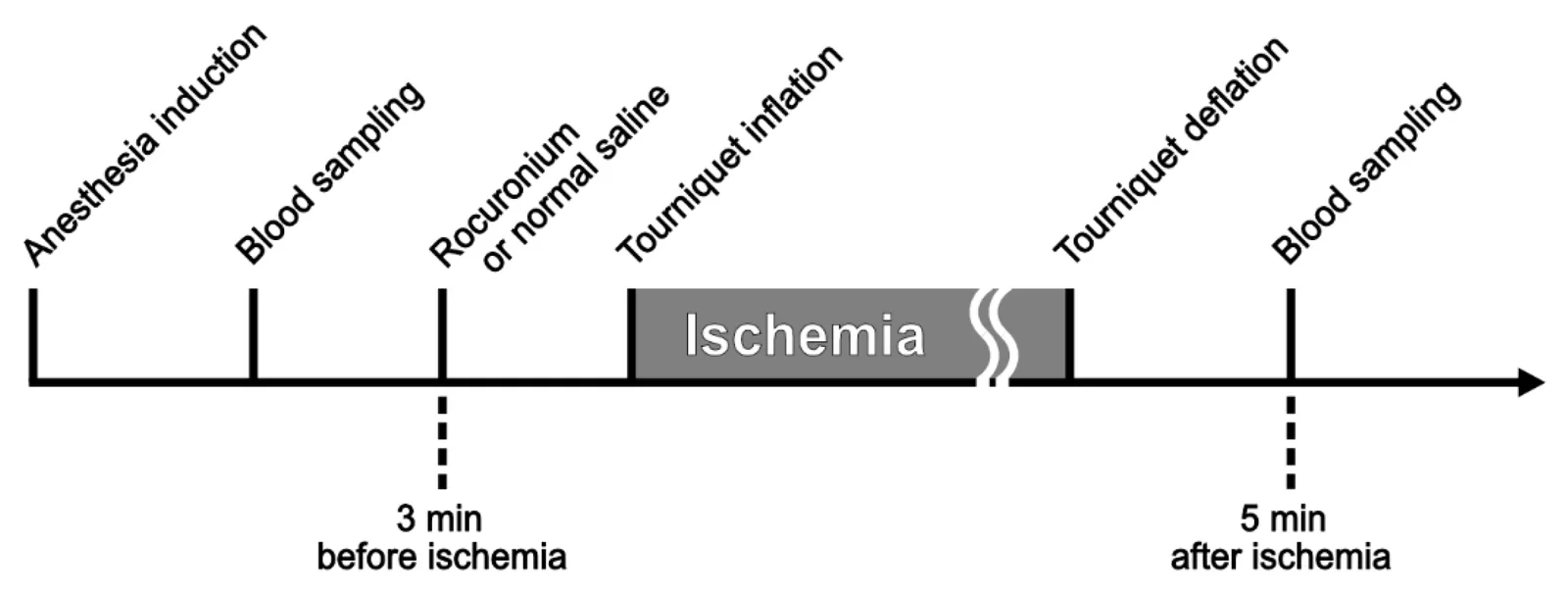
Study methods for each group

**Fig. 2:**
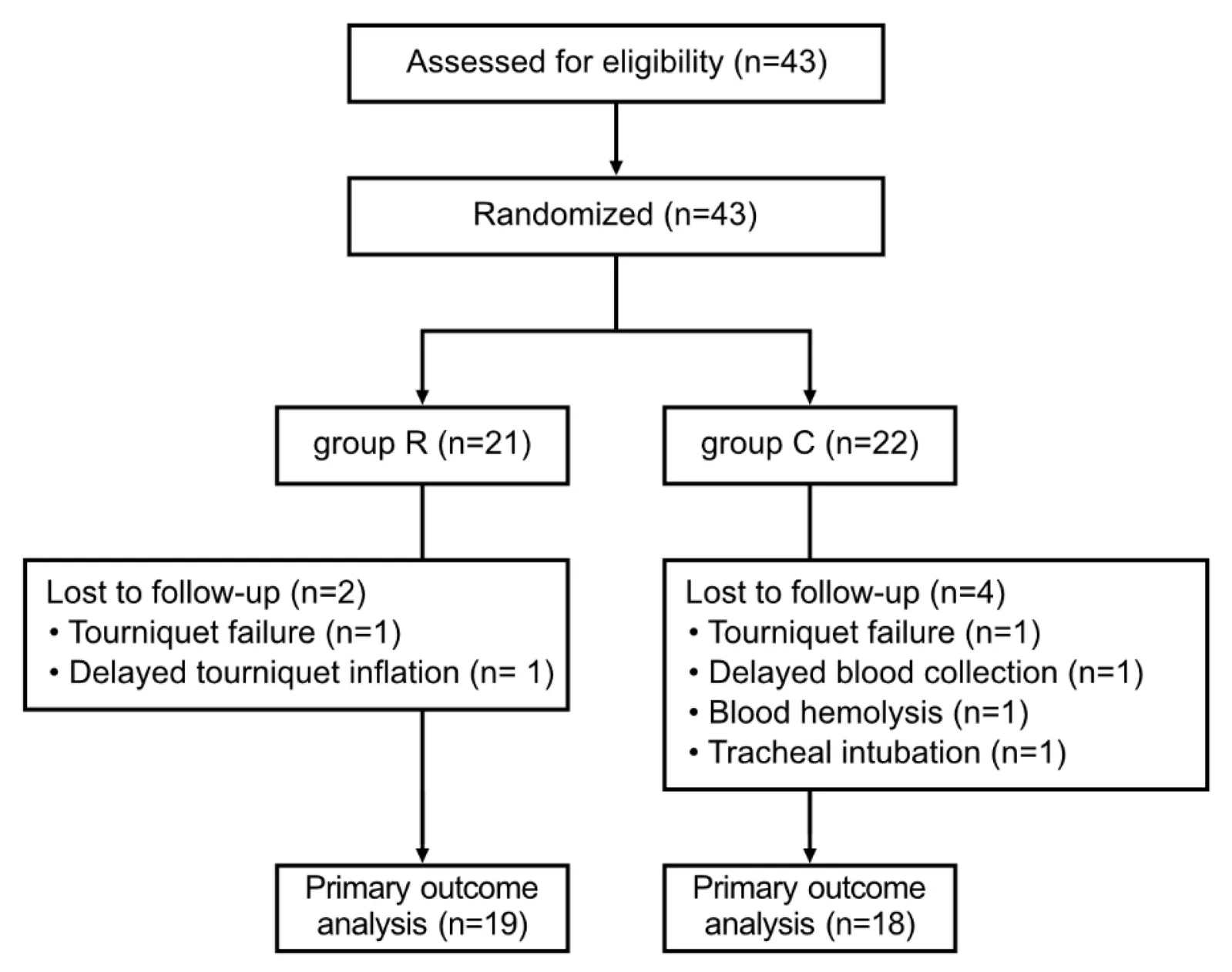
Flow chart of the study

**Fig. 3:**
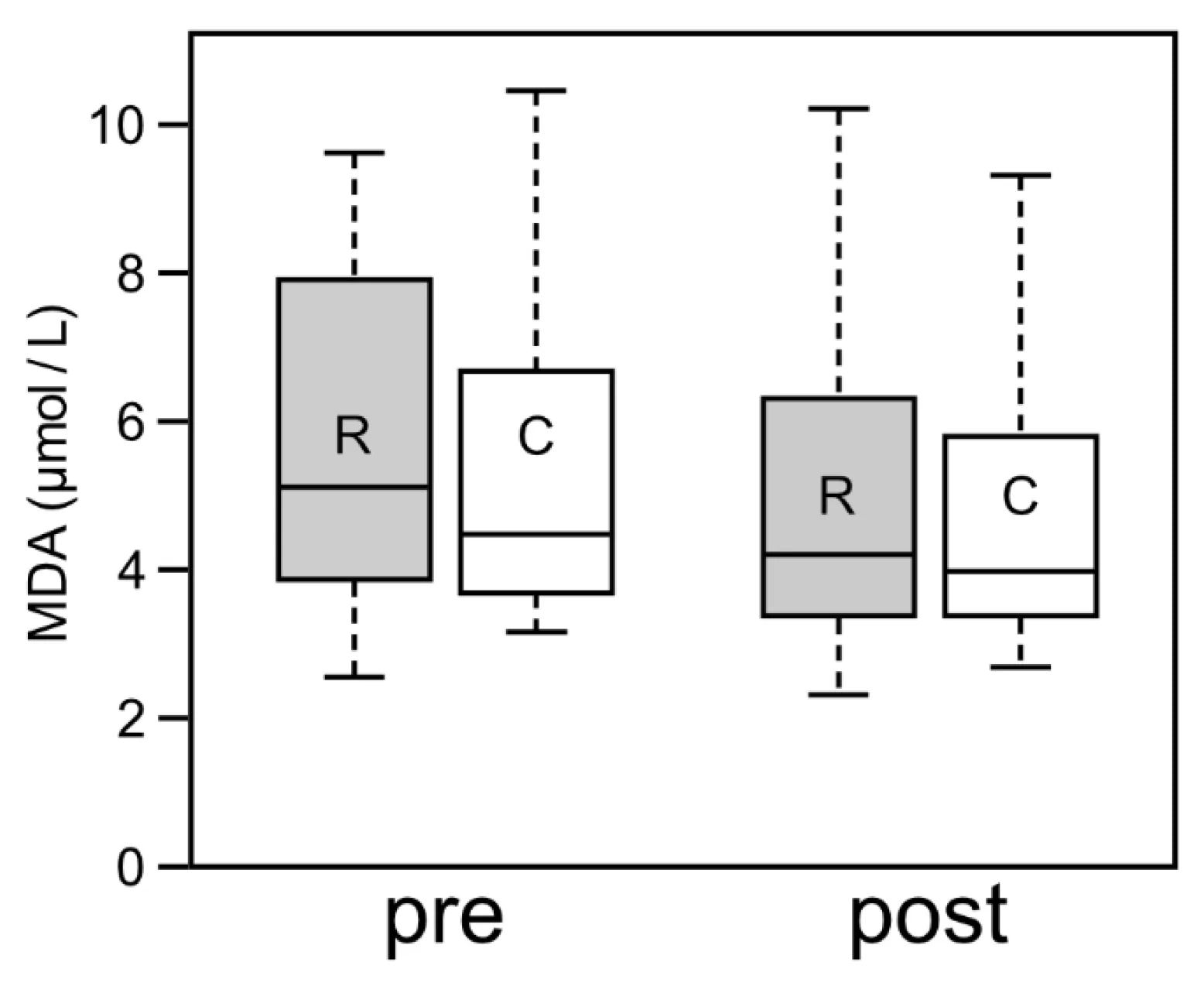
Plasma MDA concentrations before and after ischemia The box plot shows the median, interquartile range (IQR), and range of values within 1.5 times the IQR. The line inside the box represents the median. R, Rocuronium group (n = 19); C, control group (n = 18).

**Fig. 4:**
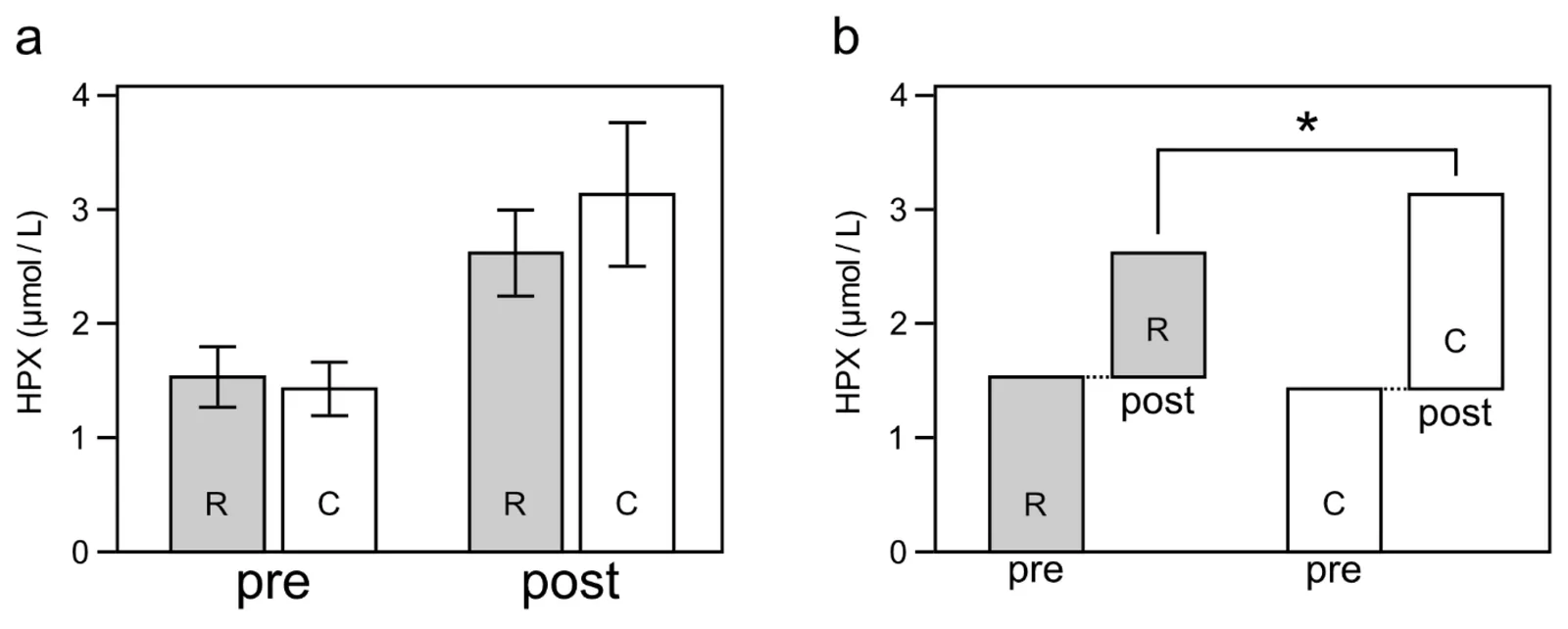
Plasma HPX concentrations before and after ischemia **a**, Comparison of concentrations. Data are presented as means with 95% confidence intervals. **b**, Comparison of concentration changes. R, Rocuronium group (n = 19); C, control group (n = 18).

**Fig. 5:**
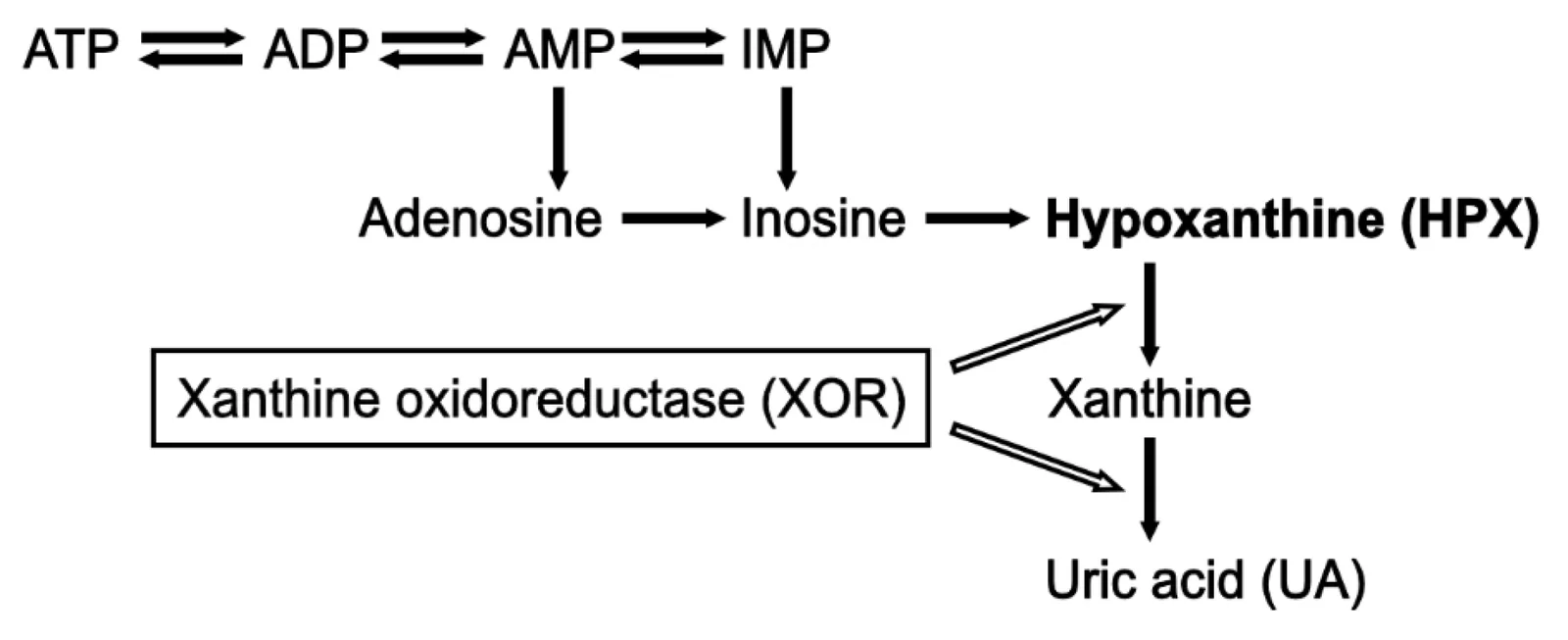
Pathway for the degradation of adenosine triphosphate (ATP) to uric acid (UA)

**Appendix 4:**
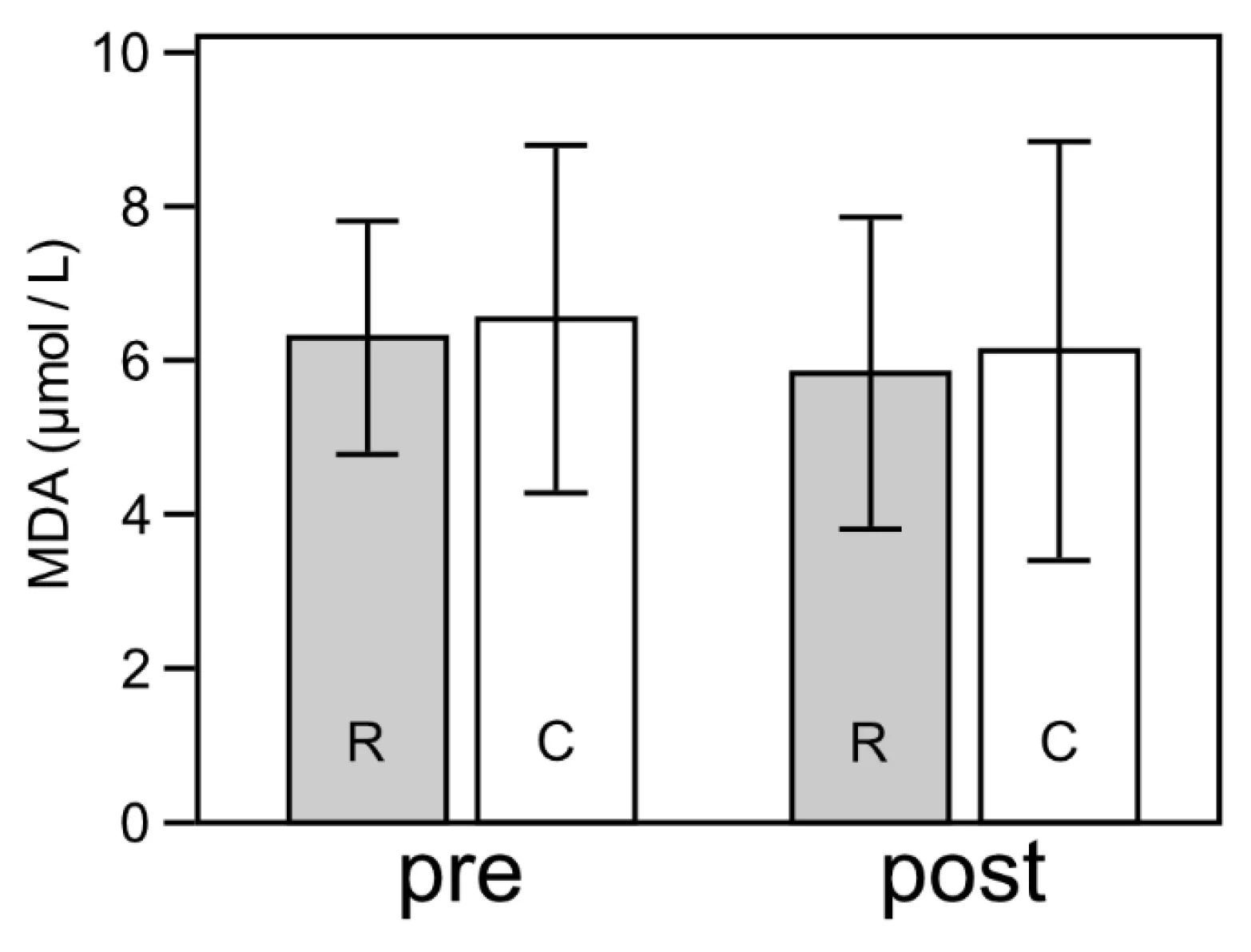
Plasma MDA concentrations before and after ischemia Data are presented as means with 95% confidence intervals. R, Rocuronium group (n = 19); C, control group (n = 18).

**Appendix 6:**
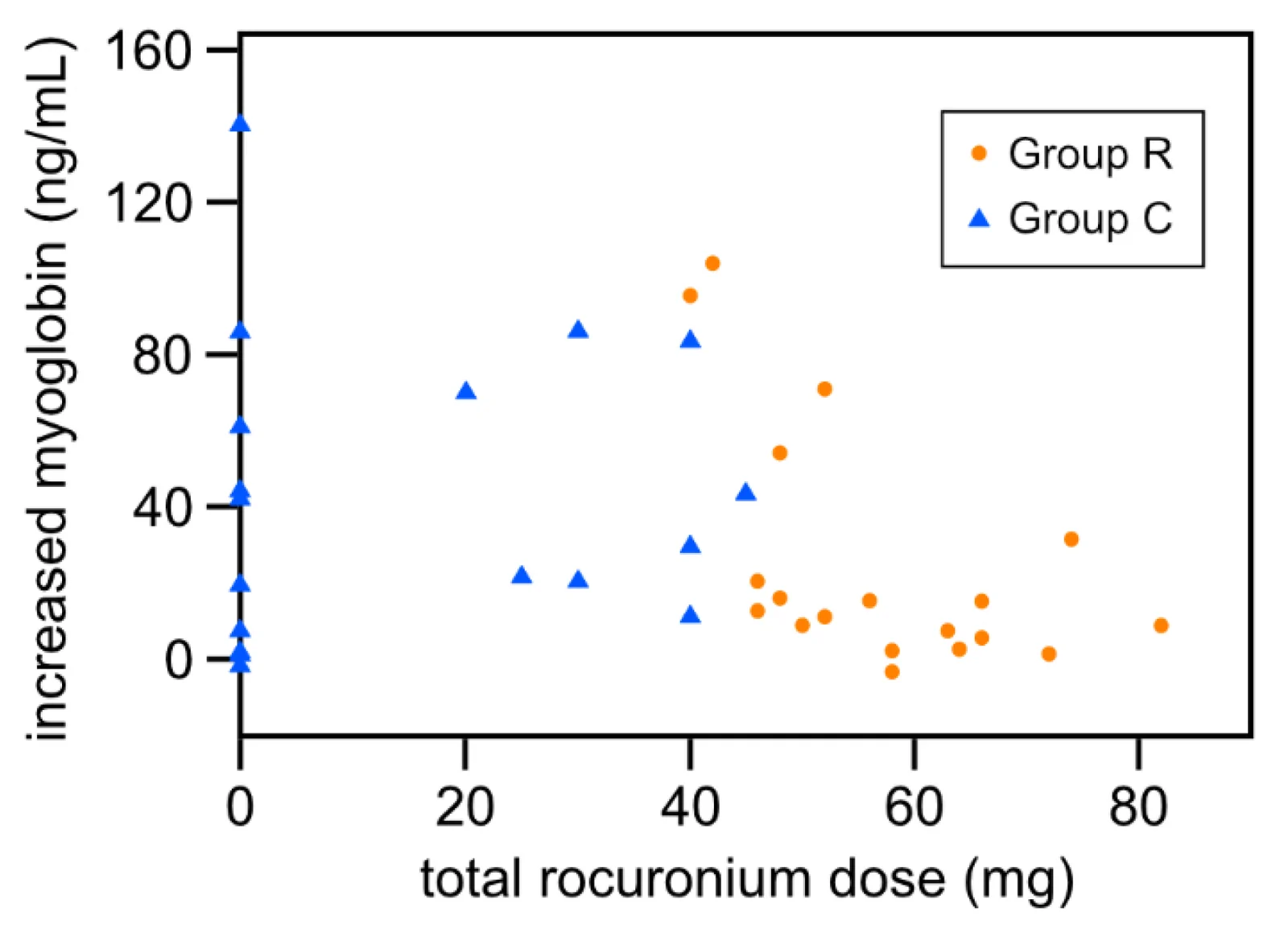
Relationship between the total rocuronium dose and increased myoglobin concentration

**Table I:** Patient demographic data

	group R (n = 19)	group C (n = 18)	*p value*
Age (y)	70 [62.5–75.5]	74.5 [70.3–77]	0.157
Height (cm)	155.1 [147.6–159.4]	151.2 [146.7–152.7]	0.071
Weight (kg)	58.3 [52.7–69.1]	54.8 [51.9–57.7]	0.145
BMI (kg/m2)	24.5 [22.5–27.7]	24.7 [22.4–26.3]	0.715
ASA-PS (I/II)	2/17	1/17	1
Operation time (min)	75 [64.5–98.5]	95.5 [67.3–112.8]	0.287
Tourniquet time (min)	74 [61.5–93]	93.5 [65–108.8]	0.186
Fluid volume (mL)	700 [615–825]	750 [625–800]	0.927
Urine output (mL)	125 [50–270]	72.5 [57.5–123.8]	0.330
Blood loss (mL)	0 [0–0]	0 [0–0]	0.938

Data are presented as medians [IQR] or numbers. We used the Mann-Whitney *U* test for continuous data and the chi-square test for categorical data.

BMI, body mass index; ASA-PS, American Society of Anesthesiologists physical status; IQR, interquartile range.

**Table II:** Primary and secondary outcomes of the study

	group R (n = 19)	group C (n = 18)	*p value*
ΔMDA (μmol/L)	−0.47 [−1.25 to 0.13]	−0.58 [−0.67 to −0.28]	0.796
ΔHPX (μmol/L)	1.09 ± 0.70	1.71 ± 1.09	0.046
ΔLactate ion (mmol/L)	0.52 ± 0.29	0.59 ± 0.29	0.418
ΔMyoglobin (ng/mL)	12.7 [6.6–26.1]	35.7 [13.1–67.6]	0.150
ΔCK (U/L)	−8.5 ± 9.8	−4.1 ± 10.1	0.181
ΔAST (U/L)	−2.0 [−4.0 to −1.0]	−2.5 [−3.8 to −1.3]	0.759
ΔPotassium (mEq/L)	0.16 ± 0.20	0.13 ± 0.25	0.690
ΔUA (mg/dL)	0 [−0.1 to 0]	0 [−0.1 to 0]	0.552

Parametric data are presented as means ± SD and non-parametric data as medians [IQR]. The Student’s *t*-test was used for parametric data and the Mann-Whitney *U* test for non-parametric data.

MDA, malondialdehyde; HPX, hypoxanthine; CK, creatine kinase; AST, aspartate aminotransferase; UA, uric acid; SD, standard deviation; IQR, interquartile range.

**Table III:** Results on quadriceps muscle strength and POD until discharge

	group R (n = 16)	group C (n = 15)	*p value*
ΔQuadriceps muscle strength (N)	−82.3 ± 43.2	−54.3 ± 47.1	0.094
POD until measurement	15 [14.8–16.3]	16 [15–17]	0.452
POD until discharge	16.7 ± 2.5	16.8 ± 2.9	0.909

Parametric data are presented as means ± SD and non-parametric data as medians [IQR]. We used the Student’s *t*-test for parametric data and the Mann-Whitney *U* test for non-parametric data.

POD, postoperative days; SD, standard deviation; IQR, interquartile range.

**Appendix 1:** Patient demographics

	group R (n = 19)	group C (n = 18)
Age (y)	67.9 ± 8.9 [51–79]	72.3 ± 6.7 [56–79]
Height (cm)	154.2 ± 7.1 [143.1–166.7]	150.2 ± 5.0 [139.8–159.2]
Weight (kg)	60.6 ± 10.0 [43.5–80]	55.5 ± 6.9 [46.5–73]
BMI (kg/m2)	25.4 ± 3.5 [21.2–32.4]	24.8 ± 3.8 [18.4–33.0]
Operation time (min)	80.2 ± 25.2 [35–123]	91.3 ± 29.3 [34–151]
Tourniquet time (min)	77.1 ± 22.7 [36–117]	87.8 ± 25.7 [36–120]
Fluid volume (mL)	718.4 ± 154.9 [400–1000]	748.3 ± 225.7 [450–1500]
Urine output (mL)	188.7 ± 179.6 [25–635]	104.4 ± 69.0 [30–250]
Blood loss (mL)	1.6 ± 6.9 [0–30]	8.9 ± 37.7 [0–160]

Data are presented as means ± SD [minimum–maximum].

BMI, body mass index; SD, standard deviation.

**Appendix 2:** Comparison of drug usage

	group R (n = 19)	group C (n = 18)	*p value*
rocuronium (mg)	56 [48–65]	0 [0–30]	<0.001
propofol (mg)	150 [120–155]	120 [120–137.5]	0.172
fentanyl (μg)	200 [150–225]	225 [200–250]	0.126
remifentanil (μg)	1507 [1040–1657]	1551 [1322–1893]	0.523
sevoflurane (mL)	21.7 [18.3–27.0]	23.1 [19.0–26.4]	0.843
ephedrine (mg)	8 [4–14]	12 [9–19]	0.102
phenylephrine (mg)	0 [0–0]	0 [0–0]	0.304
atropine (mg)	0 [0–0]	0 [0–0]	0.304
acetaminophen (mg)	1000 [1000–1000]	1000 [1000–1000]	0.645
flurbiprofen (mg)	0 [0–0]	0 [0–0]	0.341
sugammadex (mg)	130 [120–150]	0 [0–110]	<0.001
droperidol (mg)	1.25 [1.00–1.25]	1.25 [0.31–1.25]	0.768
metoclopramide (mg)	0 [0–0]	0 [0–0]	0.304
nicardipine (mg)	0 [0.0–0.7]	0 [0–0]	0.250
dexamethasone (mg)	0 [0–0]	0 [0–0]	0.141

Data are presented as medians [IQR]. *P* values were calculated using the Mann-Whitney *U* test.

IQR, interquartile range.

**Appendix 3:** Results on primary and secondary outcomes of the study

group	mean ± SD	95% CI	*p value*	median [IQR]	MIN	MAX
ΔMDA (μmol/L)						

R	−0.46 ± 2.32	−1.58 to 0.66	0.06	−0.47 [−1.25 to 0.13]	−6.15	5.53
C	−0.41 ± 2.12	NA	<0.01	−0.58 [−0.67 to −0.28]	−4.66	5.69

ΔHPX (μmol/L)						

R	1.09 ± 0.70	0.75–1.43	0.18	0.94 [0.58–1.37]	0.12	2.58
C	1.71 ± 1.09	1.17–2.25	0.50	1.70 [0.70–2.56]	0.18	4.01

ΔLactate ion (mmol/L)						

R	0.52 ± 0.29	0.38–0.66	0.29	0.50 [0.3–0.65]	0.1	1.2
C	0.59 ± 0.29	0.45–0.73	0.28	0.65 [0.4–0.8]	0.0	1.1

ΔMyoglobin (ng/mL)						

R	25.3 ± 32.0	NA	<0.01	12.7 [6.6–26.1]	−3.2	104
C	42.5 ± 38.5	23.4–61.6	0.08	35.7 [13.1–67.6]	−2.1	140

ΔCK (U/L)						

R	−8.5 ± 9.8	−13.2 to −3.8	0.09	−8.0 [−12.0 to −5.0]	−36	11
C	−4.1 ± 10.1	−9.1 to 0.9	0.73	−3.5 [−7.5 to 0.5]	−28	15

ΔAST (U/L)						

R	−1.6 ± 3.9	NA	<0.01	−2.0 [−4.0 to −1.0]	−7	12
C	−2.8 ± 3.3	NA	0.01	−2.5 [−3.8 to −1.3]	−13	3

ΔPotassium (mEq/L)						

R	0.16 ± 0.20	0.06–0.26	0.06	0.1 [0.05–0.2]	−0.1	0.6
C	0.13 ± 0.25	0.01–0.25	0.47	0.1 [0.0–0.3]	−0.3	0.7

ΔUA (mg/dL)						

R	−0.02 ± 0.09	NA	0.02	0 [−0.1 to 0]	−0.2	0.1
C	−0.04 ± 0.09	NA	0.01	0 [−0.1 to 0]	−0.2	0.1

*P* values were calculated using the Shapiro-Wilk test.

R, Rocuronium group (n = 19); C, control group (n = 18); SD, standard deviation; CI, confidence interval; IQR, interquartile range; MIN, minimum; MAX, maximum; NA, not applicable; MDA, malondialdehyde; HPX, hypoxanthine; CK, creatine kinase; AST, aspartate aminotransferase; UA, uric acid.

**Appendix 5:** Relationships between blood test results and the total rocuronium dose

	total (n = 37)	*r value*
ΔMDA (μmol/L)	−0.55 [−1.24 to −0.09]	0.09
ΔHPX (μmol/L)	1.14 [0.61–2.10]	−0.12
ΔLactate ion (mmol/L)	0.6 [0.3–0.8]	−0.07
ΔMyoglobin (ng/mL)	19.2 [7.5–54.3]	−0.31
ΔCK (U/L)	−6 [−12 to −2]	−0.38
ΔAST (U/L)	−2 [−4 to −1]	0.19
ΔPotassium (mEq/L)	0.1 [0.0–0.3]	−0.04
ΔUA (mg/dL)	0.0 [−0.1 to 0.0]	0.07

Data are presented as medians [IQR]. *R* values were calculated using Spearman’s correlation analysis.

MDA, malondialdehyde; HPX, hypoxanthine; CK, creatine kinase; AST, aspartate aminotransferase; UA, uric acid; IQR, interquartile range.

**Appendix 7:** Results on quadriceps muscle strength and POD until discharge

group	mean ± SD	95% CI	*p value*	median [IQR]	MIN	MAX
ΔQuadriceps muscle strength (N)						

R	−82.3 ± 43.2	−105.3 to −59.3	0.469	−79.5 [−118 to −53]	−140	14
C	−54.3 ± 47.1	−80.4 to −28.2	0.596	−55 [−89.5 to −12]	−123	26

POD until measurement						

R	15.1 ± 2.6	13.7–16.5	0.071	15 [14.8–16.3]	10	21
C	15.6 ± 3.1	NA	0.022	16 [15–17]	10	23

POD until discharge						

R	16.7 ± 2.5	15.4–18.0	0.102	16 [16–18]	13	23
C	16.8 ± 2.9	15.2–18.4	0.105	17 [16–18]	11	24

*P* values were calculated using the Shapiro-Wilk test.

R, Rocuronium group (n = 16); C, control group (n = 15); SD, standard deviation; CI, confidence interval; IQR, interquartile range; MIN, minimum; MAX, maximum; NA, not applicable; POD, postoperative days.
